# Inferring new indications for approved drugs via random walk on drug-disease heterogenous networks

**DOI:** 10.1186/s12859-016-1336-7

**Published:** 2016-12-23

**Authors:** Hui Liu, Yinglong Song, Jihong Guan, Libo Luo, Ziheng Zhuang

**Affiliations:** 1 Changzhou NO. 7 People’s Hospital, Changzhou, Jiangsu, 213011 China; 2 Shanghai Key Lab of Intelligent Information Processing, School of Computer Science, Fudan University, Shanghai, 200433 China; 3 Changzhou University, Jiangsu, 213164 China; 4 Department of Computer Science and Technology, Tongji University, Shanghai, 201804 China

**Keywords:** Drug positioning, Random walk, Heterogenous network

## Abstract

**Background:**

Since traditional drug research and development is often time-consuming and high-risk, there is an increasing interest in establishing new medical indications for approved drugs, referred to as drug repositioning, which provides a relatively low-cost and high-efficiency approach for drug discovery. With the explosive growth of large-scale biochemical and phenotypic data, drug repositioning holds great potential for precision medicine in the post-genomic era. It is urgent to develop rational and systematic approaches to predict new indications for approved drugs on a large scale.

**Results:**

In this paper, we propose the two-pass random walks with restart on a heterogenous network, TP-NRWRH for short, to predict new indications for approved drugs. Rather than random walk on bipartite network, we integrated the drug-drug similarity network, disease-disease similarity network and known drug-disease association network into one heterogenous network, on which the two-pass random walks with restart is implemented. We have conducted performance evaluation on two datasets of drug-disease associations, and the results show that our method has higher performance than six existing methods. A case study on the Alzheimer’s disease showed that nine of top 10 predicted drugs have been approved or investigational for neurodegenerative diseases. The experimental results show that our method achieves state-of-the-art performance in predicting new indications for approved drugs.

**Conclusions:**

We proposed a two-pass random walk with restart on the drug-disease heterogeneous network, referred to as TP-NRWRH, to predict new indications for approved drugs. Performance evaluation on two independent datasets showed that TP-NRWRH achieved higher performance than six existing methods on 10-fold cross validations. The case study on the Alzheimer’s disease showed that nine of top 10 predicted drugs have been approved or are investigational for neurodegenerative diseases. The results show that our method achieves state-of-the-art performance in predicting new indications for approved drugs.

## Background

With the increasing population age, the incidence rate of cancer is rising up and becoming a worldwide threat to human health [1–3], which leads to increasing need for anticancer drugs. However, the research and development of anticancer drugs are time-consuming and costly tasks. In recent years, many researchers and pharmaceutical enterprises turned their attentions to finding new medical indications for approved drugs [4], referred to as drug positioning or drug repurposing, because it provides a relatively low-cost and high-efficiency approach for drug discovery [5]. Nevertheless, most successfully repositioned drugs up to date have been the consequence of incidental observations of unexpected efficacy and side effects of the drugs in development or on the market [6]. It is urgent to develop rational and systematic approaches to find new indications for approved drugs on a large scale.

The explosive growth of large-scale genomic and phenotypic data, as well as the chemical and bioactivity data of thousands of compounds and natural products, allow us to develop computational methods for drug repositioning [5]. In fact, a number of computational methods have been proposed [7–10]. These methods roughly fall into three categories: machine learning, literature mining and network-based analysis [9]. Most machine learning-based methods take randomly generated drug-disease associations as negative samples, in which some false negatives are included and lead to biased decision boundary [7, 11]. The literature mining methods depend on term co-occurrence and sematic inference of some keywords of interest to infer new drug-disease associations [10, 12]. Due to the ambiguity in nature of natural language and limited accuracy of text mining techniques, literature mining-based methods do not obtain desirable performance.

Under the hypothesis that similar drugs would hold potential therapy for diseases with similar pathogenesis and symptoms, some network-based methods have been proposed to find new indications for approved drugs, by exploiting the topological and structural properties of complex biomedical networks [8, 13]. For example, Lee et al. built an integrated drug-protein-disease tripartite network, PharmDB, and proposed a so-called shared neighborhood scoring (SNS) algorithm to find new indications of known drugs [14]. Martinez et al. have proposed a network-based prioritization method, DrugNet, which integrated the information of diseases, drugs and targets to perform drug-disease and disease-drug prioritization simultaneously [15]. Chen et al. formulated the drug-disease association prediction problem as recommending preferable diseases for drugs so that two existing recommendation methods, ProbS and HeatS, were used to infer drug-disease associations [4]. Yu et al. used protein complexes as an intermediate bridge to construct a tripartite network consisting of drugs, protein complexes, and disease, on which the likelihood probabilities of drug-disease associations were inferred [16]. Luo et al. exploited known drug-disease associations to improve the drug-drug and disease-disease similarity measures, and then integrated the similarity networks and drug-disease associations to build a drug-disease heterogenous network, on which a bi-random walk algorithm is proposed to predict novel potential drug-disease associations [17]. However, current network-based methods also have some limitations. They either do not make full use of the unlabelled samples [8, 14], or are based on the predictions of two classifiers that are separately trained within the drug and disease spaces [15, 17], respectively.

In this paper, we proposed a two-pass random walk with restart on the drug-disease heterogenous network, referred to as TP-NRWRH, to predict new indications for approved drugs. The heterogenous network is built by integrating drug-drug similarity network, disease-disease similarity network and known drug-disease association network. For a candidate drug-disease association, we run two-pass random walk, a drug-centric random walk and a disease-centric random walk, to obtain the probability of arriving the objective disease node and drug node, respectively. Rather than two separate label propagation processes within the drug and disease spaces, both the drug-centric and disease-centric random walkers can travel through the whole space of the heterogenous network. The mean probabilities of the two-pass random walks are used as the confidence scores to rank all candidate drug-disease associations. We carried out performance evaluation on the widely used PREDICT dataset, and found that TP-NRWRH achieved higher performance than six existing methods on 10-fold cross validations, as well as an independent test set. On another larger dataset, our method also significantly outperformed other six competitive methods. A case study on the Alzheimer’s disease showed that nine of top 10 predicted drugs have been approved or are investigational for neurodegenerative diseases. The results show that our method achieves state-of-the-art performance in predicting new indications for approved drugs.

## Methods

### Drug-disease association network

The drug-disease association network is constructed by collecting known associations between a set of drugs and diseases of interest. The drug-disease associations are often extracted by professional biocurators from FDA-approved drug indications and biomedical publications. Formally, denote by *C*={*c*_1_,*c*_2_,…,*c*_*n*_} and *D*={*d*_1_,*d*_2_,…,*d*_*m*_} the drug and disease node set, and *A* the adjacent matrix of drug-disease association network with element *a*_*il*_=1 if there is known association between drug *i* and disease *l*, or *a*_*il*_=0 otherwise.

### Drug-drug similarity network

We compute two similarity measures for each pair of drugs based on the chemical fingerprints and known drug-disease associations, and then integrate the two similarity measures to a comprehensive measure. The first similarity measures is based on the chemical fingerprints of the drug molecules. The chemical fingerprints are generated by using the PaDEL software (release v2.21) [18], which takes as input the SMILES of the drugs to generate the chemical fingerprints, as well as many other chemical attributes. There are totally 800 kinds of chemical fingerprints, and thus each drug was represented by a 880-dimension binary vector, in which the element is equal to 1 if the corresponding chemical fingerprints is contained in the drug, or 0 otherwise. With the vector form of the chemical fingerprints, we can easily compute the Jaccard score of two drugs as the chemical similarity. The Jaccard score, which is widely used for measuring the similarity and diversity of finite sample sets, is defined as the ratio between the number of common fingerprints of two drugs to their total number of fingerprints. Let $\vec {f}_{i}$ and $\vec {f}_{j}$ be the vector forms of the chemical fingerprints of drug *c*_*i*_ and *c*_*j*_, the chemical similarity $w_{ij}^{(c1)}$ between drug *c*_*i*_ and *c*_*j*_ is defined as below:

1 $$ w_{ij}^{(c1)}=\frac{| \vec{f}_{i}\cap \vec{f}_{j} |}{|\vec{f}_{i}\cup \vec{f}_{j}|}.  $$

Besides, we can compute another drug-drug similarity measure by exploiting the known drug-disease associations. In particular, we adopt the bipartite network projection proposed by [19] to derive the strength of relatedness of two drugs. The bipartite network projection is inspired by the network-based resource-allocation dynamics, which consists of two resource transfer steps. In terms of the drug-disease bipartite network, the resource originally held by each drug node is equally distributed to its disease neighbors, and then the resource assigned to each disease node is equally distributed back to its drug neighbors. Therefore, the second drug-drug similarity, denoted by $w_{ij}^{(c2)}$, is defined as the proportion of the resource distributed from drug *c*_*i*_ to drug *c*_*j*_ during the resource allocation process. Assume each drug node initially owns one-unit resource, $w_{ij}^{(c2)}$ can be formulated as:

2 $$  w_{ij}^{(c2)} = \frac{1}{k(c_{i})}\sum\limits_{l=1}^{m}\frac{a_{il}a_{jl}}{k(d_{l})},  $$

in which *k*(*c*_*i*_) and *k*(*d*_*l*_) are the degree of drug *c*_*i*_ and disease *d*_*l*_ in the drug-disease association network. Note that this measure is not symmetrical, as $w_{ij}^{(c2)}$ is often unequal to $w_{ji}^{(c2)}$. The intuitive explanation is that more common disease neighbors of two drugs have, larger the similarity measure is. When two drugs have no common known disease, the similarity is equal to 0.

Subsequently, the two drug-drug similarities are integrated into a comprehensive similarity measure by the probability disjunction formula:

3 $$  w_{ij}^{(c)} = 1 - \left(1-w^{(c1)}_{ij}\right)\left(1-w^{(c2)}_{ij}\right),  $$

in which $w^{(c)}_{ij}$ represents the integrative similarity measure between drug *c*_*i*_ and drug *c*_*j*_.

### Disease-disease similarity network

We build disease-disease similarity network by integrating two disease-disease similarity measures derived from disease phenotypes and known drug-disease associations. The phenotype-based measure is calculated using MimMiner [20], which adopt an approach analogous to the term frequency-inverse document frequency (tf-idf) technique widely used in information retrieval to compute the phenotype similarity. More precisely, MimMiner represents each disease-related phenotype by a vector of MeSH concepts extracted from the OMIM database [21], and then computes the cosine similarity between two MeSH concept vectors. Denote by $\vec {t}_{i}=\{t_{i1},t_{i2},\ldots,t_{iK}\}$ and $\vec {t}_{j}=\left \{t_{j1},t_{j2},\ldots,t_{jK}\right \}$ the MeSH concept vectors of disease *d*_*i*_ and disease *d*_*j*_, the phenotype-based similarity $w_{ij}^{(d1)}$ is formulated as:

4 $$  w_{ij}^{(d1)} = \frac{\sum_{k=1}^{K} t_{ik}t_{jk}}{\sqrt{\sum_{k=1}^{K} t_{ik}^{2}}\sqrt{\sum_{k=1}^{K} t_{jk}^{2}}},  $$

in which *K* represents the total length of the dictionary of MeSH concepts.

Similarly, we compute another disease-disease similarity by using the bipartite network projection mentioned above. Let $w_{ij}^{(d2)}$ be the proportion of the resource distributed to disease *d*_*j*_ from drug *d*_*i*_, we have

5 $$  w_{ij}^{(d2)} = \frac{1}{k(d_{i})}\sum\limits_{l=1}^{n}\frac{a_{il}a_{jl}}{k(c_{l})}  $$

in which *k*(*d*_*i*_) and *k*(*c*_*l*_) is the degree of disease *d*_*i*_ and drug *c*_*l*_ in the drug-disease association network. The similarity $w_{ij}^{(d2)}$ between disease *d*_*i*_ and disease *d*_*j*_ has a similar intuitive explanation, i.e. more common drug neighbors of two diseases have, larger the similarity is. When two diseases have no common known drug, the similarity is equal to 0. We combine the two individual disease-disease similarities into a comprehensive similarity by using the probability disjunction formula as below:

6 $$  w_{ij}^{(d)} = 1 - \left(1-w^{(d1)}_{ij}\right)\left(1-w^{(d2)}_{ij}\right),  $$

in which $w^{(d)}_{ij}$ represents the integrative similarity between disease *d*_*i*_ and disease *d*_*j*_.

### Two-pass random walk with restart on heterogenous network

**Fig. 1:**
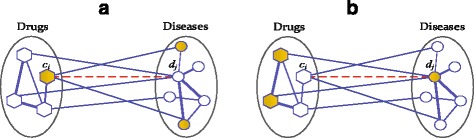
The illustrative diagram of the two-pass random walk with restart on drug-disease heterogenous network. For a candidate association between drug *c*_*i*_ and disease *d*_*j*_, a two-pass random walk process is run to compute its final confidence score. The nodes covered in the initial probability distribution are in *gold color*, and the candidate drug-disease association is represented by *dashed line*. **a** The drug-centric random walk process starts from drug *c*_*i*_ and all its known associated diseases. **b** The disease-centric random walk process starts from disease *d*_*j*_ and all known associated drugs

Based on the aforementioned drug-drug similarity network, disease-disease similarity network and drug-disease association network, we build a drug-disease heterogenous network *G*=(*V*,*E*). The node set *V*={*C*,*D*} is the union of the drug and disease node sets. The edge set *E*=*E*_*cc*_ ∪ *E*_*dd*_ ∪ *E*_*cd*_ in which *E*_*cc*_, *E*_*dd*_ and *E*_*cd*_ are the sets of drug-drug edges, disease-disease edges and drug-disease edges, respectively. Based on the drug-disease heterogenous network, we extend the network-based random walk with restart on the heterogeneous network (NRWRH) developed by [22] to infer potential drug-disease associations. For a candidate drug-disease association between drug *c*_*i*_ and disease *d*_*j*_, we run two-pass random walks with restart on the heterogenous network, a drug-centric random walk and a disease-centric random walk, to determine its confidence score. As shown in Fig. 1 a, the drug-centric random walk starts from drug *c*_*i*_ and its known associated diseases, and derive the probability of the random walker arriving at disease *d*_*j*_ when steady state is reached. Accordingly, the disease-centric random walk starts from disease *d*_*j*_ and its known associated drugs, and derive the probability of the random walker arriving at drug *c*_*i*_ when steady state is reached, as shown in Fig. 1 b. Finally, we compute the mean probability of the two-pass random walks as its confidence score. Compared to traditional NRWRH algorithm, the two-pass random walk with restart on heterogenous network, TP-NRWRH for short, effectively balances the probabilities derived from two single-pass random walks for each candidate drug-disease association (see Discussion and conclusion for more details).

If a random walker starts from a drug node on the heterogenous network *G*, it can jump to one of the associated disease nodes with probability *λ*, or jump to any other drug nodes with probability 1- *λ*. A random walker can only travel within one type of networks, if *λ*=0. Therefore, we constructed the transition matrix *T* as

7 $$ T=\left[ \begin{array}{cc} T^{(cc)} & T^{(cd)} \\ T^{(dc)} & T^{(dd)} \\ \end{array} \right]  $$

where *T* ^(*cc*)^ and *T* ^(*dd*)^ are transition matrix of the probability from one drug (disease) to other drug (disease) in the random walk, respectively; *T* ^(*cd*)^ is the transition matrix from drug network to disease network, and *T* ^(*dc*)^ is the transition matrix from disease network to drug network. Based on the drug-drug similarity defined in Eq. (3), the transition probability from drug *c*_*i*_ to drug *c*_*j*_ is defined as

$$T^{(cc)}_{ij}= \left\{ \begin{array}{ll} w_{ij}^{(c)}/\sum_{k=1}^{n} w_{ik}^{(c)}, & \text{if } \sum_{l=1}^{m} a_{il}=0, \\ (1-\lambda)w_{ij}^{(c)}/\sum_{k=1}^{n} w_{ik}^{(c)}, & \text{otherwise}. \end{array}\right. $$

Similarly, the transition probability from disease *d*_*i*_ to disease *d*_*j*_ can be defined on the basis of the disease-disease similarity defined in Eq. (6). Formally, the transition probability from disease *d*_*i*_ to disease *d*_*j*_ is defined as

$$T^{(dd)}_{ij}= \left\{ \begin{array}{ll} w_{ij}^{(d)}/\sum_{k=1}^{m} w_{ik}^{(d)}, & \text{if } \sum_{l=1}^{n} a_{li}=0, \\ (1-\lambda)w_{ij}^{(d)}/\sum_{k=1}^{m} w_{ik}^{(d)}, & \text{otherwise}. \end{array}\right. $$

The transition probability from drug *c*_*i*_ to disease *d*_*j*_ is defined as

$$T^{(cd)}_{ij}= \left\{ \begin{array}{ll} \lambda a_{ij}/\sum_{l=1}^{m} a_{il}, & \text{if } \sum_{l=1}^{m} a_{il}\neq0, \\ 0, & \text{otherwise}. \end{array}\right. $$

Similarly, the transition probability from disease *d*_*i*_ to drug *c*_*j*_ is defined as

$$T^{(dc)}_{ij}= \left\{ \begin{array}{ll} \lambda a_{ji}/\sum_{l=1}^{n} a_{li}, & \text{if } \sum_{l=1}^{n} a_{li}\neq0, \\ 0, & \text{otherwise}. \end{array}\right. $$

Let *P*(*t*) be a (*n*+*m*)-dimension vector in which the *i*-th element represents the probability of finding the random walker at node *i* at step *t*, the probability can be calculated iteratively by

8 $$ P(t+1)=(1-\alpha)T^{\prime}P(t)+\alpha P_{0},  $$

where *α* is the restart probability at each step, and *P*_0_ is the initial probability distribution over some given seed nodes. For drug-centric random walk, a specific drug and its known associated diseases are regarded as seed nodes, as shown in Fig. 1 a. Take drug *c*_*i*_ as an example, *c*_*i*_ is denoted as the seed node in the drug network and given probability 1, while other nodes in the drug network are given probability 0. In this way, we construct the initial probability regarding the drug nodes. Besides, the disease nodes associated to drug *c*_*i*_ are regarded as seed nodes in disease network and given equal probabilities so that the sum of their probabilities is equal to 1, forming the initial probability regarding the disease nodes. Denote by $P_{0}^{(c)}$ and $P_{0}^{(d)}$ the initial probabilities regarding the drug and disease nodes, we define the initial probability *P*_0_ for drug-centric random walk as

9 $$ P_{0}=\left[ \begin{array}{c} \eta P_{0}^{(c)}\\ (1-\eta)P_{0}^{(d)}\\ \end{array} \right],  $$

in which the parameter *η*∈ [ 0,1] is a tradeoff factor to balance the weight of importance between the drug network and target network. Similarly, we can construct the initial probability distribution for disease-centric random walk. As shown in Fig. 1 b, *d*_*j*_ is denoted as the seed node in the disease network and given probability 1, other nodes in the disease network are given probability 0, forming the initial probability $P_{0}^{(d)}$ regarding disease nodes. The drug nodes associated to disease *d*_*j*_ are used as seed nodes in the drug network and given equal probabilities so that the sum of their probabilities is equal to 1, forming the initial probability $P_{0}^{(c)}$ regarding drug nodes. As a result, the initial probability *P*_0_ for disease-centric random walk is formulated as

10 $$ P_{0}=\left[ \begin{array}{c} (1-\eta)P_{0}^{(c)}\\ \eta P_{0}^{(d)}\\ \end{array} \right].  $$

Let *P* ^∗^ be the vector when the random walks converge, i.e. the change between *P*(*t*) and *P*(*t*+1) (measured by the L1 norm) is less than a very small number *ε* (=1.0E-10), $P^{*}_{i}$ is the probability of finding the random walker at node *i* in the steady state. Once the two-pass random walks for a candidate drug-disease association are finished, the mean probability is computed as its confidence score, which is used to rank all candidate drug-disease associations.

## Results

### Competitive methods used in performance evaluation

To evaluate the performance of the proposed method, we compare it with six existing methods on two different datasets. Two methods, MBiRW [17] and DrugNet [15], have been proposed to predict drug-disease associations. Four other methods, including NBI [23], HGBI [24], KBMF2K [25] and DT-Hybrid [26], have been originally developed for predicting drug-target interactions but are applicable in the prediction of drug-disease associations. MBiRW exploits known drug-disease associations to improve the drug-drug and disease-disease similarity measures, and then integrates the similarity networks and drug-disease associations to build a drug-disease heterogenous network on which a bi-random walk algorithm is proposed to predict novel potential drug-disease associations [17]; DrugNet is a network-based drug repositioning method, which is able to perform both drug-disease and disease-drug prioritization [15]; NBI predicts new drug-target interactions by running a two-step diffusion model on the drug-target bipartite graph [23]; HGBI is based on the guilt-by-association principle and predict new drug-target associations by iteratively updates the measure of strength between unlinked drug-target pairs by taking all the paths in the network into account [24]; KBMF2K uses kernelized bayesian matrix factorization with twin kernels to predict drug-target interactions [25]; DT-Hybrid extends the NBI algorithm by adding domain knowledge including drug-drug similarity and target-target similarity into the original model.

In particular, each method is configured to its default setting or best parameter values reported in its paper. In particular, the parameters (*λ*,*α*,*η*) included in TP-NRWRH are set to (0.8, 0.3, 0.4) in following experiments. MBiRW is run in its default setting, namely, the restart probability *α* is 0.3 and the numbers of maximal iterations in the left and right random walks are equal to 2. For DrugNet, the restart probability *α* is set to its default value 0.3. For HGBI, both the restart probability *α* and the cutoff for drug-drug and disease-disease connections are set to their best values 0.4 and 0.3, respectively. For KBMF2K, we use KBMF2K-classification model and kept its default parameter values. The two parameters *α* and *λ* included in DT-Hybrid are set to the reported values 0.7 and 0.8, as these values are used in the original paper.

### Evaluation on PREDICT dataset

We first carry out performance evaluation on a drug-disease association dataset published by Gottlieb et al. [27]. The dataset is manually curated from multiple resources and published in accompany with a novel computational method called PREDICT for predicting new drug indications [27]. For convenience, we refer to this dataset as PREDICT dataset in the following experiments. The PREDICT dataset includes 1933 known drug-disease associations involving 593 approved drugs in DrugBank [28] and 313 diseases in the Online Mendelian Inheritance in Man (OMIM) [21].

#### 10-fold cross validations

We conduct 10-fold cross-validations on the PREDICT dataset to compare the performance of our TP-NRWRH and other six existing methods. The drug-disease associations in PREDICT dataset are randomly split into 10 subsets with roughly equal size, and then each subset is taken in turn as a test set and the remaining nine subsets are taken as input to run our method. The prediction accuracies are calculated on the test subset, and the averages over the 10-fold test subsets are regarded as overall performance measures.

**Fig. 2:**
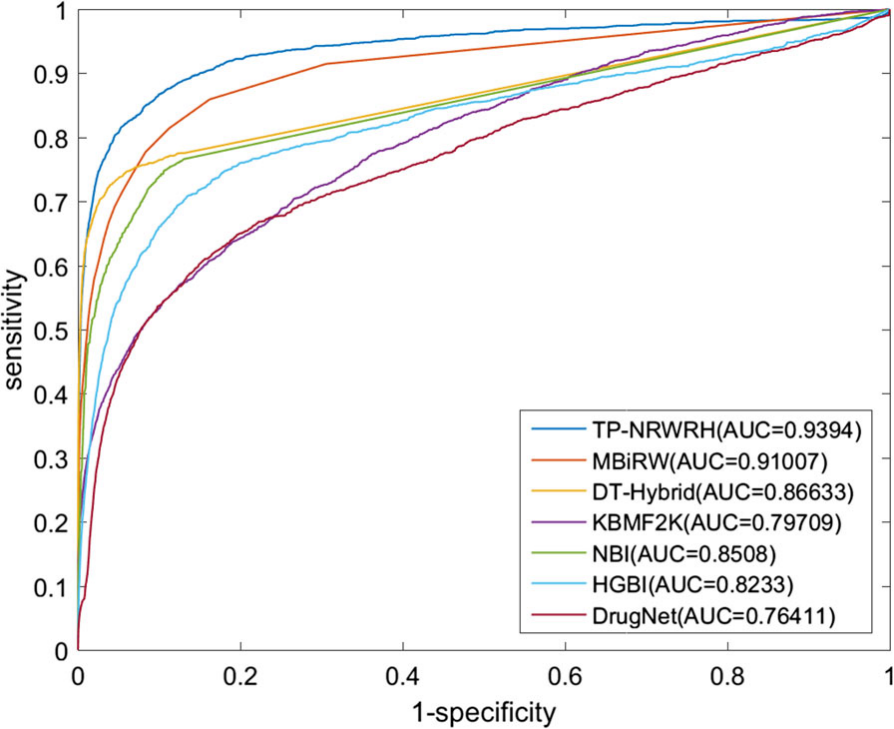
The ROC curves and AUC values of the proposed TP-NRWRH and six existing methods on the PREDICT dataset

The ROC curves of TP-NRWRH and other six methods on the PREDICT dataset are shown in Fig. 2. It can be found that TP-NRWRH significantly outperforms all other competitive methods. TP-NRWRH achieves the highest AUC 0.9394, followed by MBiRW at 0.9134 AUC value. The performance of DrugNet is the worst and gets only 0.7641 AUC value.

**Fig. 3:**
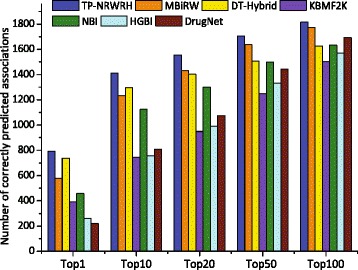
The number of correctly predicted drug-disease associations by our method and six existing methods on the PREDICT dataset, with respect to five different top-ranked thresholds

Since the number of correctly predicted true positives reflects the discriminatory power of a prediction method to distinguish true positives, especially when the number of negative samples is far larger than that of positive samples. Therefore, we report the number of correctly predicted drug-disease associations with respect to a specified top-rank threshold. A known drug-disease association is considered as correctly predicted if its ranking according to the predicted confidence score is higher than a specified top-rank threshold. As shown in Fig. 3, we report the number of correctly predicted drug-disease associations by the seven methods for top 1, 10, 20, 50 and 100 rank thresholds. It can be seen that our method correctly predicts more true drug-disease associations than other six methods upon each top-rank threshold.

#### Evaluation on independent test set

For objective performance evaluation, another dataset released by [17] is used to assess the performance of the seven methods. By removing the drugs not included in PREDICT, we produce an independent test set including 89 drug-disease associations regarding 71 drugs and 313 diseases. Here, we use it to assess the performances of the seven prediction methods, by predicting the drug-disease associations based on the PREDICT dataset and calculating the performance measures on the independent test set.

**Fig. 4:**
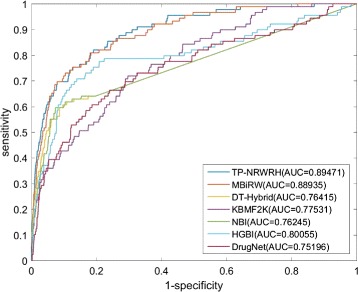
The ROC *curves* and AUC values of TP-NRWRH and six existing methods on the independent test set. Note that the predictions are based on PREDICT dataset, while the performance measures are calculated on the independent test set

**Fig. 5:**
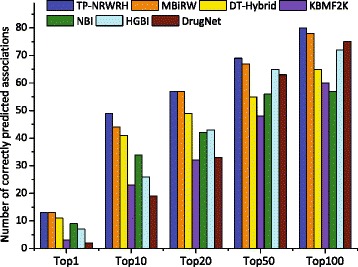
The number of correctly predicted drug-disease associations by TP-NRWRH and six existing methods on the independent test set, with respect to five different top-ranked thresholds

The ROC curves of the seven competitive methods on the independent test set are shown in Fig. 4. Overall, the performance of all the methods moderately deteriorate relative to the 10-fold cross validations. TP-NRWRH still holds the highest performance by achieving 0.8947 AUC value. MBiRW and HGBI successively follow our method by 0.8893 and 0.8006 AUC values, while the AUC values of the remaining four methods are no less 0.8. We also show the number of correctly predicted drug-disease associations with respect to given top-ranked thresholds, as shown in Fig. 5. Accordingly, TP-NRWRH achieves more correctly predicted drug-disease associations than all other six methods on almost every top-rank threshold except top 50.

### Evaluation on Cdataset

**Fig. 6:**
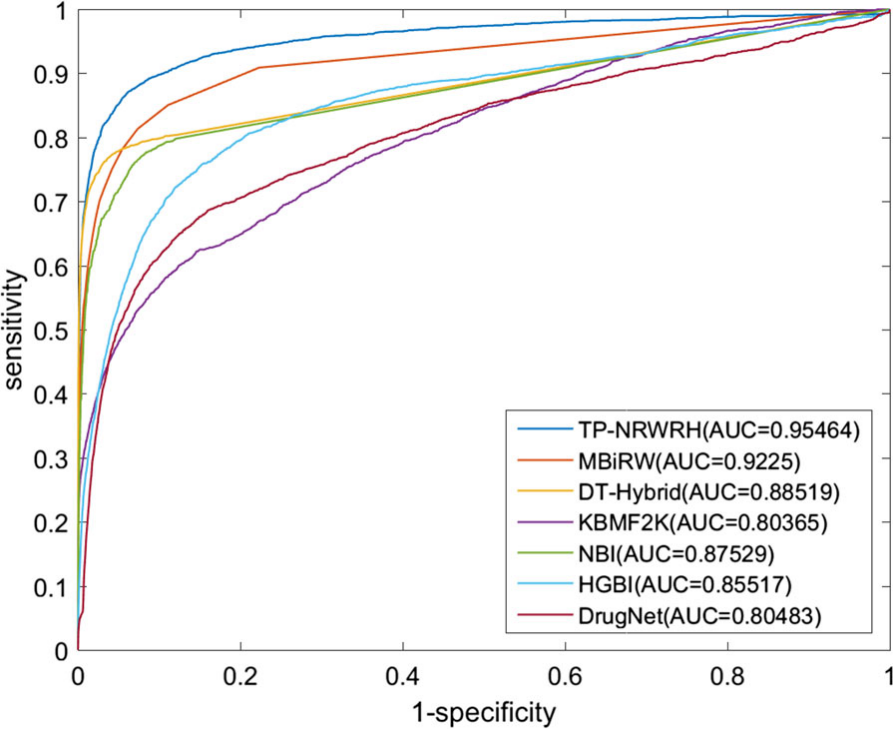
The ROC curves and AUC values of TP-NRWRH and six existing methods on the Cdataset

**Fig. 7:**
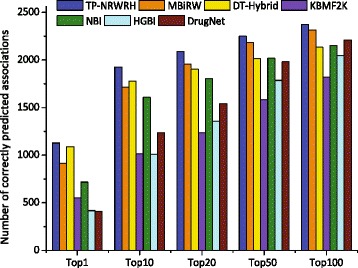
The number of correctly predict drug-disease associations by TP-NRWRH and six existing methods on the Cdataset, with respect to three different top-ranked thresholds

We further evaluate the performance of the proposed method on another larger dataset than PREDICT dataset, referred to as Cdataset, which is published by Luo et al. [17]. The Cdataset includes 2,352 known drug-disease associations between 663 drugs and 409 diseases. Similarly, ten-fold cross validations are conducted to compare the performance of the seven competitive methods, and the results are shown in Fig. 6. It can be seen that TP-NRWRH obtains the AUC value 0.9546, which is significantly higher than that of other six competitive methods. MBiRW still closely follows our method on Cdataset by 0.9225 AUC value. Interesting, the performance of each method notably rise up on Cdataset compared to PREDICT dataset. In terms of the number of correctly predicted drug-disease associations, TP-NRWRH has the best performance on every top-rank threshold, as shown in Fig. 7.

### Case study: Alzheimer’s disease

To further validate the performance of the proposed method, we conduct a case study for Alzheimer’s disease. We report the top 10 predicted drugs for Alzheimer’s disease, as shown in Table 1. For each drug, we show the canonical name and DrugBank Accession Number, drug-centric probability score, disease-centric probability score and mean probability. Through retrieval of DrugBank, we have found that nine of the10 drugs, except for Calcitriol, are muscarinic antagonists or antimuscarinics-like agents that have been approved or investigational for neurodegenerative diseases such as Parkinson’s disease. In despite of the difference in pathogenesis between Parkinson’s disease and Alzheimer’s disease, they are common neurodegenerative diseases associated with aging [29]. Moreover, a recent study has revealed that Parkinson’s disease and Alzheimer’s disease are genetically related, as both diseases are primarily caused by deposits of some common proteins in the brain. There are certain strains of the alpha-synuclein protein associated with Parkinson’s disease that can encourage the accumulation of the tau protein associated with Alzheimer’s [30]. More interestingly, the drug Calcitriol is an active form of vitamin D(3) metabolite and a receptor in the central nervous system. Calcitriol have been suggested to play beneficial role in improving the cognitive function in some patients with Alzheimer’s disease [31, 32]. These previous findings strongly support the predicted drugs are potential indications for Alzheimer’s disease.

**Table 1:** Top 10 predicted drugs for Alzheimer’s disease by TP-NRWRH

Drug name	DrugBank ID	Drug-centric prob.	Disease-centric prob.	Mean prob.
Biperiden	DB00810	0.010013127	0.0027618815	0.006387
Procyclidine	DB00387	0.007374576	0.0029763145	0.005175
Benzatropine	DB00245	0.007368236	0.0029541662	0.005161
Carbidopa	DB00190	0.005865933	0.0030864640	0.004476
Ropinirole	DB00268	0.005859384	0.0030558408	0.004458
Pramipexole	DB00413	0.005862381	0.0030442238	0.004453
Scopolamine	DB00747	0.003635959	0.0033643315	0.003500
Calcitriol	DB00136	0.003123367	0.0014964786	0.002310
Trihexyphenidyl	DB00376	0.003490107	0.0005885250	0.002039
Bromocriptine	DB01200	0.003481560	0.0005622120	0.002022

## Discussion and conclusion

**Fig. 8:**
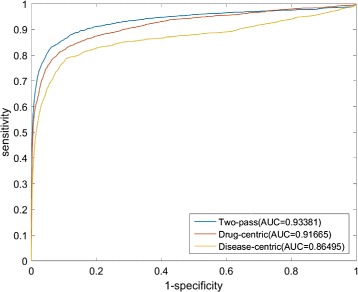
The ROC curves and AUC values of TP-NRWRH (two-pass) and the two single-pass NRWRH, drug-centric and disease-centric algorithms, on the PREDICT dataset

In this paper, we propose a network-based method to predict new indications for approved drugs. To verify the performance of the proposed method, we use several network-based methods for predicting drug-target interactions and drug-disease associations in our empirical experiments. In fact, our method is inspired by the network-based random walk with restart on heterogenous network (NRWRH) [22], which run only drug-centric random walk with restart on drug-target heterogenous network to predict new targets for a drug of interest. To test whether the two-pass NRWRH (TP-NRWRH) really improves the performance of traditional NRWRH, we conduct another experiment to compare the performance of TP-NRWRH and two single-pass NRWRH, i.e. drug-centric and disease-centric random walks on heterogenous network, on the PREDICT dataset. The experimental results are shown in Fig. 8, it can be found that TP-NRWRH significantly outperforms the drug-centric and disease-centric algorithms. We postulate that the drug-centric and disease-centric random walks are actually asymmetric label propagation processes, which would provide complementary information for a candidate drug-disease association, while TP-NRWRH gracefully balances the probabilities derived from the two single-pass random walks and thus achieves better performance.

Our another concern is that the network topological structure of the heterogenous network may affect the performance of our method. Especially, the existences of the edges linking drugs and diseases depend on the collected drug-disease associations. However, current collection of drug-disease associations is often incomplete, and the strengths of the associations between drugs and diseases are actually quantitative. We suggest that quantitative associations rather than qualitative associations between heterogenous nodes probably improve the performance of our method, and we thus plan to verify this point in our future work.

We have conducted empirical experiments to compare the performance of TP-NRWRH and other six popular methods on two different datsets. One the PREDICT dataset, a widely used standard dataset in drug positioning, TP-NRWRH achieved higher performance than six existing methods on both the 10-fold cross validations and an independent test set. On another larger dataset, our method also significantly outperforms the other six competitive methods. Moreover, the case study on the Alzheimer’s disease showed that nine of the top 10 predicted drugs have been approved for neurodegenerative diseases. The results show that our method achieves state-of-the-art performance for the discovery of new drug-disease associations.
